# Parsonage-Turner Syndrome

**DOI:** 10.5334/jbsr.3088

**Published:** 2023-04-28

**Authors:** Elyn Van Snick, Bjorn Valgaeren, Bart Claikens

**Affiliations:** 1 Vrije Universiteit Brussel, BE; 2 KUL, BE; 3 AZ Damiaan, BE

**Keywords:** Parsonage-Turner syndrome, denervation, infraspinatus muscle, magnetic resonance imaging

## Abstract

**Teaching Point:** Magnetic resonance imaging is a valuable imaging tool in Parsonage-Turner syndrome, a rare neurological disorder that presents as acute denervation in the distribution of the brachial plexus.

## Case History

**Figure 1:**
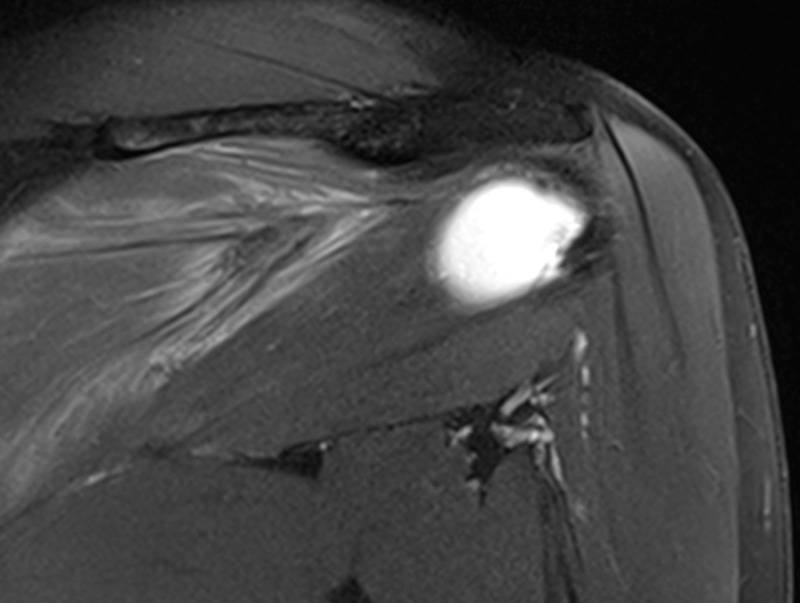


**Figure 2:**
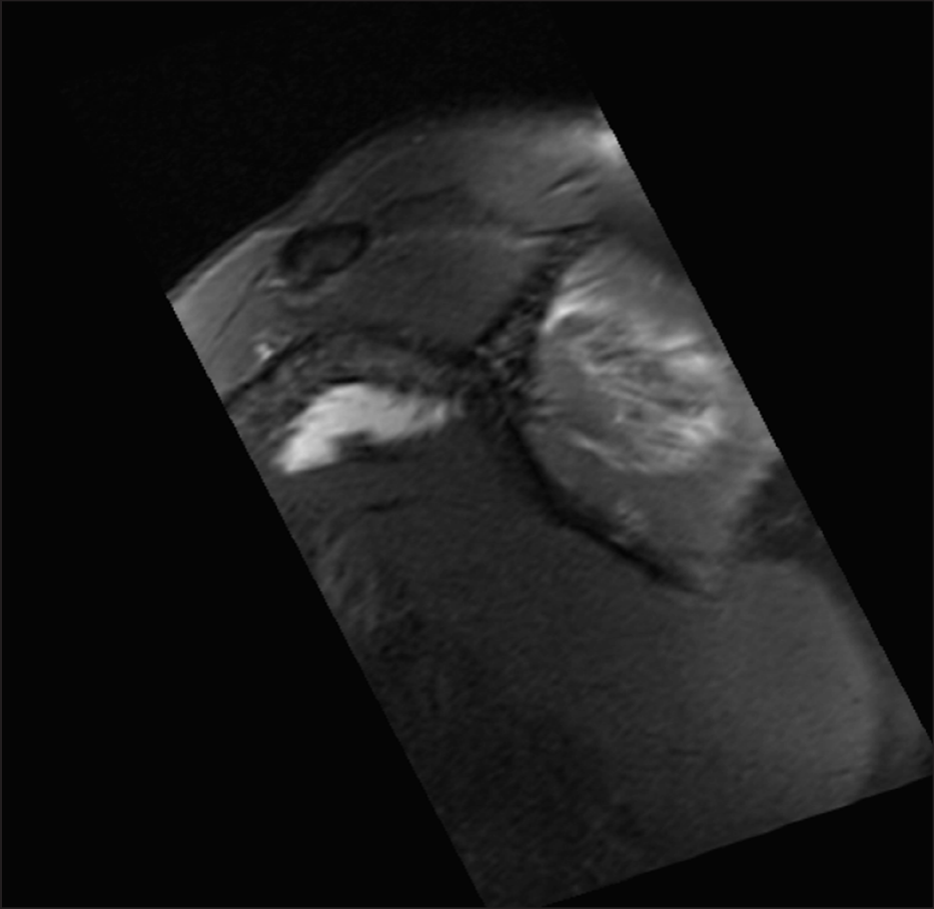


A 57-year-old man consulted the orthopaedic surgeon because of atraumatic left shoulder pain that had been present for over five months. He experienced activity-related pain and had trouble carrying out overhead movements. Clinical examination revealed limited shoulder mobility and slightly diminished force exerted by the infraspinatus muscle. A magnetic resonance imaging (MRI) arthrography was performed, which showed tendinopathy and a partial thickness tear of the supraspinatus tendon. Also, T2 weighted images with fat supression (FS T2-WI) showed hyperintense oedematous infiltration of the infraspinatus muscle (Figures 1 and 2).

Electromyography (EMG) showed isolated neuropathy of the suprascapular nerve with signs of denervation of the infraspinatus muscle. There were no signs of external compression of the suprascapular nerve on the MRI exam.

**Figure 3:**
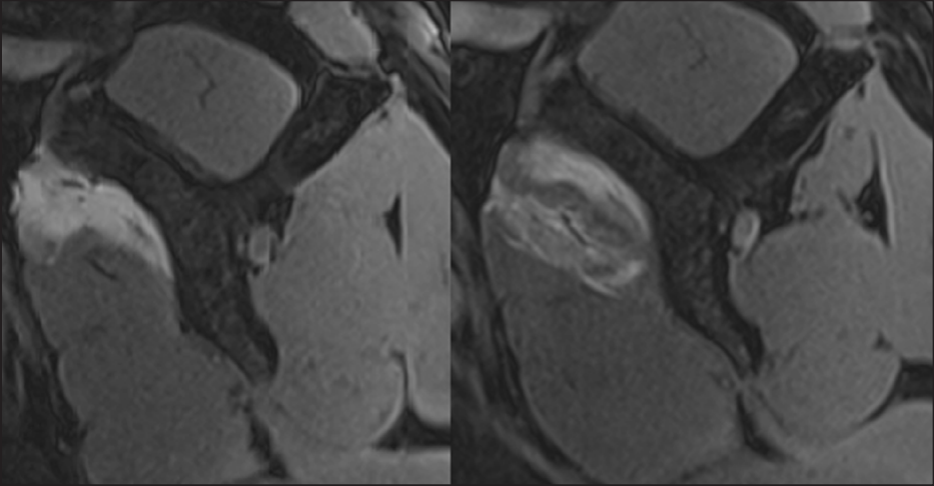


Treatment by means of physiotherapy and vitamin B supplementation was initiated. MRI examination nine months later showed resolution of the oedematous changes in the infraspinatus muscle with minor muscle atrophy and fat infiltration of the infraspinatus muscle belly (Figure 3). Infraspinatus muscle strength on clinical examination and EMG findings had normalized. The combination of history, EMG results, and imaging findings is compatible with Parsonage-Turner syndrome.

It is a bit unusual for the patient to present with chronic pain, as Parsonage-Turner syndrome typically manifests as acute onset of severe shoulder pain and/or muscle weakness, but this can probably be attributed to his concomitant rotator cuff disease. It is unclear why only the infraspinatus muscle was involved, since the affected suprascapular nerve also innervated the supraspinatus muscle.

## Comments

Parsonage-Turner syndrome is a rare neurological disorder, also known as ‘acute idiopathic brachial neuritis’ [1]. Patients present with acute shoulder pain and/or progressive muscle weakness and paraesthesia. The causative mechanism is not fully understood but viral and autoimmune processes have been suggested to play a role. The disorder has a male predilection and age at presentation varies widely. Most cases are unilateral, although bilateral involvement has been reported in up to one out of three cases.

Diagnosis is made by a combination of clinical history, EMG results, and imaging findings. EMG may show signs of acute denervation in the distribution of the brachial plexus, with the suprascapular nerve being most frequently affected. The suprascapular nerve is a branch of the superior trunk of the brachial plexus, innervating the supraspinatus and infraspinatus muscles.

MRI is a sensitive tool in demonstrating signal abnormalities in the shoulder musculature that reflect denervation. These signal abnormalities can vary widely and change depending on the stage of the disease. In the early acute phase, the signal intensity of the muscles may be normal on MRI. The earliest sign of denervation visible on MRI is an increase in T2 signal intensity reflecting muscular edema, which usually develops at about two weeks after the symptom onset. Muscular edema is best appreciated on fluid-sensitive sequences, which are more sensitive for the detection of muscular edema than conventional T2-WI sequences. The increase in T2-weighted signal may occur with or without an accompanying change in T1-weighted signal. In the subacute phase, T2-weighted signal abnormalities persist and muscular atrophy and T1-hyperintense fatty infiltration may start to develop. This can progress rather quickly over the course of 2-5 weeks. Muscular atrophy and fatty infiltration are best evaluated on T1-WI without FS (T1-weighted imaging without fat saturation), preferentially in the oblique sagittal plane. The chronic phase is characterized by muscular atrophy and fatty infiltration and lasts several weeks or months, after which signal abnormalities may disappear. In some patients, however, there is no resolution of the abnormalities.

MRI is also useful to detect structural abnormalities, such as spinoglenoid and suprascapular notch masses that may compress the nerve, and to rule out more common causes of shoulder pain. Direct MR arthrography as performed in the case may be helpful in the evaluation of rotator cuff disease, a common cause of shoulder pain, but can render evaluation of muscular edema more difficult due to contrast leakage in the muscles and is therefore not recommended when Parsonage-Turner syndrome is suspected. However, as was the case in our patient, findings suggestive of Parsonage-Turner syndrome are often found on MRI exams performed for other reasons.

Parsonage-Turner syndrome is typically self-limiting and clinical symptoms and abnormalities on MRI may disappear after a few months. Treatment consists of analgesics and physiotherapy.
